# María Teresa Anguera Argilaga: pioneer of observational methodology and an example of wisdom and humanity

**DOI:** 10.3389/fpsyg.2025.1617013

**Published:** 2025-06-06

**Authors:** Antonio Hernández-Mendo, José Luis Losada

**Affiliations:** ^1^University of Malaga, Málaga, Spain; ^2^University of Barcelona, Barcelona, Catalonia, Spain

**Keywords:** observational metodology, mixed methods, program assessment, qualitative analysis, observational designs

María Teresa Anguera Argilaga, professor emeritus at the University of Barcelona, dedicated her life to developing observational methodology in behavioral sciences and to humbly and generously training those who knew her. Her legacy leaves a deep mark on education, psychology, and social sciences.

At a time when Spanish psychology was asserting its scientific identity, Dr. Anguera built a solid multidisciplinary foundation by studying teaching, philosophy (with a specialization in psychology), and law, thus enriching her scientific vision. She stood out for her methodological rigor, her keen analytical skills, and, fundamentally, for her human qualities and commitment to service.

## A career of academic excellence and multidisciplinary application

Dr. Anguera's research focused on observational methodology and program evaluation design, leading numerous competitive projects and publishing in prestigious international journals. Her integrative approach to qualitative and quantitative methods made her a key figure in the development of *Mixed Methods*.

She was principal investigator on multiple initiatives and coordinated the Research and Innovation in Designs Group (GRID), promoting the application of multimedia and digital technologies in research. Her contributions, such as the innovative HOISAN software, made it possible to code and analyze behavior with unprecedented accuracy. These tools have facilitated the study of social and behavioral dynamics in classrooms and sports environments.

Her work has been especially valuable in providing practical methods for understanding and evaluating human interactions. Her ability to connect theory and practice inspired generations of professionals to approach their disciplines with scientific rigor and human sensitivity.

## Exceptional teacher, inspiring mentor, and model of humility

In her teaching role as professor in the Department of Behavioral Science Methodology at the University of Barcelona, Dr. Anguera taught at all educational levels. Her commitment to education transcended the classroom, actively participating in doctoral and master's programs at universities in Spain and Portugal, always with a clear, rigorous, and approachable teaching style.

However, what truly distinguished Dr. Anguera was her profound humility and generous willingness to help others. Despite her many achievements, she always remained accessible and altruistic. Students and colleagues remember her dedication to guiding those who sought her advice, both academically and personally. Her professional ethics and human warmth made her an exemplary role model, both in the academic and personal spheres.

For her students, she was a constant source of inspiration, teaching them that academic excellence must be inseparably linked to empathy and social commitment, fundamental values for those dedicated to educating others.

## Well-deserved recognition and an indelible legacy

Her outstanding career was recognized with honorary doctorates from universities such as La Laguna, Las Palmas de Gran Canaria, Pontificia de Salamanca, and Lérida. However, her greatest legacy lies in the transformative impact of her work and the example she set for us: the harmonious combination of academic excellence, deep empathy, and dedication to service.

Dr. Anguera's passing left an irreplaceable void in the Spanish Academy of Psychology (of which she was a member) and in the university, but also an indelible legacy. Her smile, her kindness, and her constant willingness to help live on in the memory of those who knew her. Her influence transcended research methodology; she transformed the understanding of science and the meaning of serving others. She was a teacher of methodology and, even more, a teacher of life, demonstrating that professional success and humility not only can but must coexist.

For future professionals and researchers, her life remains a timeless testament to the idea that excellence is not defined solely by academic accolades, but by the ability to inspire and elevate others. Her legacy lives on through the countless applications of Observational Methodology and the classrooms shaped by her pedagogical influence. She revolutionized the way we teach, investigate, and understand science with precision and humanity.

Beyond her scholarly legacy, Dr. Anguera embodied the rare blend of intellectual rigor and human warmth. Her journey reminds us that true success lies in cultivating both professional excellence and an unwavering commitment to the service of others—with empathy, humility, and gratitude.

